# Isolating and Characterizing the Translatome From Human Alzheimer's Disease (AD) Brains

**DOI:** 10.1192/bjo.2024.113

**Published:** 2024-08-01

**Authors:** Muhammad Ali Bangash, Julie Qiaojin Lin

**Affiliations:** 1 Department of Old Age Psychiatry, Kings College, London, United Kingdom; 2 South London and Maudsley NHS Trust, London, United Kingdom; 3 Dementia Research Institute, Cambridge, United Kingdom; 4 Hong Kong University of Science and Technology, Guangzhou, China

## Abstract

**Aims:**

Local protein synthesis at the synapse is a key determinant of learning and memory and is predicted to be severely disrupted in Alzheimer's disease (AD). Omics approaches have played a key role in deciphering molecular mechanisms underlying AD pathology. However, isolating the transcriptome may be biased due to inherent variations in transcript levels, or by transcription-on-demand models employed by several genes, whereas mass-spec based proteomics approaches fail to capture low abundance peptides. The translatome bypasses these inherent limitations of other omics methods by capturing actively translating mRNA species trapped inside ribosomes and subjecting them to unbiased RNA-seq analysis capturing even very low abundance transcripts.

**Methods:**

Isolating the neuronal ribosomes from human post-mortem brains without interference from non-neuronal cells remains a challenge. We used frozen brain tissue from Alzheimer's patients and healthy controls obtained from the Cambridge Brain Biobank. Synaptoneurosomal fractions were prepared using sucrose gradients in non-denaturing buffers with RNAse inhibitors to preserve ribosomal composition and trapped mRNA. We isolated functional ribosomes on affinity columns following recombinant RNAse digestion. Finally, actively translating ribosome-trapped mRNAs were sequenced using RNA-seq, aligned to human genome using STAR alignment and analysed for differential expression using DeSeq2 followed by pathway analysis.

**Results:**

We have successfully isolated ribosome-associated RNA transcripts in the dendritic spines from cortical neurons of postmortem Alzheimer's brains with little interference from glial and non-neuronal material. The novel AD translatome disruptions identified by isolating endogenous ribosome bound mRNA will help detect downstream molecular targets. We will also integrate targeted translatome data with published transcriptome and GWAS DNA variant data to identify novel biomarkers.

**Conclusion:**

This is the first successful isolation of the dendritic translatome from human postmortem AD brains. Future studies will verify functional significance of key targets using gain- and loss-of-function studies in animal models of AD and human iPSCs.

